# Mortality in ST-Segment Elevation Myocardial Infarction With Nonobstructive Coronary Arteries and Mimickers

**DOI:** 10.1001/jamanetworkopen.2023.43402

**Published:** 2023-11-16

**Authors:** Odayme Quesada, Mehmet Yildiz, Timothy D. Henry, Seth Bergstedt, Jenny Chambers, Ananya Shah, Larissa Stanberry, Lucas Volpenhein, Dalia Aziz, Rebekah Lantz, Cassady Palmer, Justin Ugwu, Muhammad J. Ahsan, Ross F. Garberich, Heather S. Rohm, Frank V. Aguirre, Santiago Garcia, Scott W. Sharkey

**Affiliations:** 1 Women’s Heart Center, The Christ Hospital Heart and Vascular Institute, Cincinnati, Ohio; 2 The Carl and Edyth Lindner Center for Research and Education, The Christ Hospital, Cincinnati, Ohio; 3 Minneapolis Heart Institute Foundation at Abbott Northwestern Hospital, Minneapolis, Minnesota; 4 Prairie Heart Institute at St John’s Hospital, Springfield, Illinois; 5 Iowa Heart Center, Des Moines

## Abstract

**Importance:**

The clinical characteristics and prognosis of patients with ST-segment elevation myocardial infarction (STEMI) with nonobstructive coronaries (MINOCA) are largely unknown.

**Objective:**

To assess differences in 5-year mortality in patients presenting with STEMI due to MINOCA and MINOCA mimickers as compared with obstructive disease.

**Design, Setting, and Participants:**

A retrospective analysis of a prospective registry-based cohort study of consecutive STEMI activations at 3 regional Midwest STEMI programs. STEMI without a culprit artery and elevated troponin levels were categorized as MINOCA (absence of coronary artery stenosis >50% and confirmed or suspected coronary artery plaque disruption, epicardial coronary spasm, or coronary embolism/thrombosis) or MINOCA mimickers (takotsubo cardiomyopathy, myocarditis, or nonischemic cardiomyopathy). Data were analyzed from March 2003 to December 2020.

**Main Outcomes and Measures:**

Adjusted Cox regression analysis was used to assess 5-year mortality risk in STEMI presenting with MINOCA and MINOCA mimickers in comparison with obstructive disease.

**Results:**

Among 8560 consecutive patients with STEMI, mean (SD) age was 62 (14) years, 30% were female (2609 participants), and 94% were non-Hispanic White (4358 participants). The cohort included 8151 patients with STEMI due to obstructive disease (95.2%), 120 patients with MINOCA (1.4%), and 289 patients with MINOCA mimickers (3.8%). Patients were followed up for a median (IQR) of 7.1 (3.6-10.7) years. Patients with MINOCA and MINOCA mimickers were less likely to be discharged with cardiac medications compared with obstructive disease. At 5-year follow-up, mortality in STEMI presenting with obstructive disease (1228 participants [16%]) was similar to MINOCA (20 participants [18%]; χ^2^_1_ = 1.1; log-rank *P* = .29) and MINOCA mimickers (52 participants [18%]; χ^2^_1_ = 2.3; log-rank *P* = .13). In adjusted Cox regression analysis compared with obstructive disease, the 5-year mortality hazard risk was 1.93 times higher in MINOCA (95% CI, 1.06-3.53) and similar in MINOCA mimickers (HR, 1.08; 95% CI, 0.79-1.49).

**Conclusions and Relevance:**

In this large multicenter cohort study of consecutive clinical patients with STEMI, presenting with MINOCA was associated with a higher risk of mortality than obstructive disease; the risk of mortality was similar in patients with MINOCA mimickers and obstructive disease. Further investigation is necessary to understand the pathophysiologic mechanisms involved in this high-risk STEMI population.

## Key Points

**Question:**

Is 5-year mortality different in patients with ST-segment elevation myocardial infarction (STEMI) presenting with nonobstructive coronaries (MINOCA) and MINOCA mimickers (takotsubo cardiomyopathy, myocarditis, or nonischemic cardiomyopathy) as compared with patients with obstructive disease?

**Findings:**

In this cohort study of 8560 consecutive patients with STEMI, compared with obstructive disease, 5-year mortality hazard risk was higher in patients with MINOCA and similar in patients with MINOCA mimickers.

**Meaning:**

The findings of this study suggest that STEMI without obstructive disease is a morbid disease, emphasizing the need to diagnose the underlying cause of MINOCA and MINOCA mimickers at the time of the event.

## Teaser

This cohort study evaluates mortality in ST-segment elevation myocardial infarction with nonobstructive coronary arteries.

## Introduction

Among patients presenting with acute myocardial infarction (AMI), the culprit artery cannot be identified in 5% to 15% of cases.^1,2,3^ The majority of AMI cases, including ST-segment elevation myocardial infarction (STEMI) and non-STEMI (NSTEMI), without any culprit artery are classified as myocardial infarction with nonobstructive coronary arteries (MINOCA).^4^

MINOCA is a syndrome composed of distinct diagnoses based on pathophysiologic mechanisms. The 2019 American Heart Association (AHA) statement defines MINOCA as coronary artery plaque disruption, epicardial coronary spasm, or coronary embolism/thrombosis and excludes takotsubo cardiomyopathy, myocarditis, and nonischemic cardiomyopathy due to their failure to meet the fourth universal definition of MI and classifies them as MINOCA mimickers.^5,6^ Our current understanding of MINOCA is limited for the following reasons: (1) the term MINOCA is used interchangeably with AMI without a culprit artery; however, not all patients without a culprit artery have nonobstructive coronary arteries; (2) inclusion of cases without troponin elevation; (3) discordance between the AHA 2019 and the European Society of Cardiology (ESC) 2016 statement, which includes MINOCA mimickers in the definition of MINOCA syndrome^6,7^; (4) lack of transparency in published studies on the MINOCA definition and diagnostic criteria used; (5) limited use of cardiac magnetic resonance imaging (CMRI) which is the reference standard diagnostic test for evaluating patients presenting with MINOCA to determine the underlying diagnosis; and (6) limited studies to date of patients with MINOCA presenting with STEMI.

MINOCA has historically been considered a low-risk AMI phenotype. However, unfavorable outcomes have been reported in patients with MINOCA compared with healthy individuals without any cardiovascular disease.^8,9,10^ When compared with obstructive coronary artery disease, some studies reported better outcomes in patients with MINOCA^11,12,13^; whereas others noted similar or even higher mortality risk in patients with MINOCA.^14,15,16^ These disparate findings are primarily due to the limitations of current MINOCA studies.

Despite worse prognosis in patients with MINOCA presenting with STEMI compared with NSTEMI,^17,18^ knowledge on the outcomes in patients with STEMI presenting with MINOCA as compared with obstructive disease is limited to 2 studies that we know of.^19,20^ Both included patients with and without elevated troponin in the MINOCA group and failed to report whether MINOCA mimickers were included in the MINOCA cohort. Therefore, we examined a large prospective multicenter US cohort of consecutive STEMI activations to evaluate the prevalence, characteristics, and 5-year mortality risk of patients with MINOCA and MINOCA mimickers in comparison with obstructive disease.

## Methods

### Study Population

The Midwest STEMI Consortium is composed of 4 high-volume, tertiary, regional STEMI centers, including Minneapolis Heart Institute in Minneapolis, Minnesota (site 1); The Christ Hospital in Cincinnati, Ohio (site 2); Prairie Heart Institute in Springfield, Illinois (site 3); and Iowa Heart Center in Des Moines, Iowa (site 4).^21^ Data from site 4 were not included in the index study, given the inability to provide 5-year mortality data. All consecutive patients beginning March 2003 who had a STEMI activation within 24 hours of symptom onset were prospectively included in the database. These regional tertiary centers use similar standardized STEMI protocols and serve as the referral centers for over 100 nonpercutaneous coronary intervention (PCI) hospitals with a spectrum of urban and rural communities. The design of the Midwest STEMI consortium has been described in detail previously.^21^ The study protocol, data sharing agreements, and relevant information have been approved by institutional review boards (IRB) in each study center. Informed consent or waiver of consent was obtained as required by each institution's IRB. The study follows the Strengthening the Reporting of Observational Studies in Epidemiology (STROBE) reporting guideline for observational studies.^22^

The study flow diagram is shown in eFigure 1 in Supplement 1. We identified a total of 10 624 consecutive STEMI activations and excluded 2064 cases. Each STEMI without a culprit artery case was retrospectively reviewed by the principal investigator at each site, including history of presentation, coronary angiogram, left ventriculogram, and echocardiogram images, and CMRI when available. Of note, intravascular imaging (intravascular ultrasound or optical coherence tomography) was not performed at any sites.

The STEMI cases without culprit artery were further categorized according to the 2019 AHA position statement as either MINOCA or MINOCA mimickers.^6^ MINOCA was defined as (1) absence of stenosis greater than 50% in any major epicardial vessels during invasive coronary angiography by visual estimation, (2) evidence of ischemia defined as at least 1 troponin measurement above the 99th percentile, and (3) absence of an alternative diagnosis at the index presentation. Diagnoses of MINOCA included confirmed or clinically suspected coronary artery plaque disruption, epicardial coronary spasm, and coronary embolism/thrombosis. Patients with spontaneous coronary artery dissection (SCAD) causing obstructive lesions were included in the obstructive disease group, and SCAD without obstructive lesion were included in the MINOCA group. MINOCA mimicker cases met the diagnostic criteria for takotsubo cardiomyopathy, nonischemic cardiomyopathy, and myocarditis as previously described.^23,24,25^

### Data Collection and Follow-Up

At the time of the STEMI event, trained research assistants collected all relevant demographic clinical data (including self-reported race), angiography, laboratory markers performed by the hospital laboratory, and discharge medications by reviewing electronic medical records. Race was included because it is an important social determinants of health that is associated with cardiovascular outcomes. The standardized protocols included serial cardiac troponin I and T assays. A positive troponin was defined as at least 1 troponin measurement above the 99th percentile for the assay-specific reference limit, which varied over time and by site. When available, the following CMRI data were collected: evidence of infarction (late gadolinium enhancement), regional wall motion abnormality, and final impression by reader.

Major adverse cardiovascular events (MACE), defined as cardiac arrest, myocardial infarction, or physician-adjudicated cardiac-related death were collected and analyzed at 1 year at sites 1 and 2. Time to MACE was defined as time to the first adverse event in the MACE definition. All-cause mortality was collected in-hospital and at 1 and 5-year follow-up at all sites from the electronic medical records and the national death index. The end of follow-up for the mortality outcome was February 2023. Only the initial STEMI event was included in the analysis and all subsequent events were excluded. There were 335 patients without follow-up data (either due to in-hospital mortality or loss to follow-up) who were censored at day 0.

### Statistical Analyses

Categorical variables are presented as numbers and relative frequencies (percentages computed out of all nonmissing data) and continuous variables as mean (SD) or median (IQR). Outcomes were compared between STEMI presenting with obstructive disease as compared with MINOCA and MINOCA mimickers. Also, outcomes between MINOCA and MINOCA mimickers were compared. Categorical variables were compared using the Fisher exact test or χ^2^ test and continuous variables using 2-sided Wilcoxon rank-sum tests or independent sample 2-sided *t* tests.

The primary end point of the study was 5-year mortality risk. Survival and MACE-free survival from the time of STEMI presentation to 1 and 5 years were estimated using the Kaplan-Meier method and compared between STEMI presenting with obstructive disease as compared with MINOCA and MINOCA mimickers using the log-rank test. In sensitivity analysis, the Kaplan-Meier method was used to estimate 5-year mortality in CMRI-confirmed MINOCA cases as compared with obstructive disease using the log-rank test.

Additionally, Cox proportional hazard models were built to estimate relative hazard rates of 5-year mortality among patients with MINOCA and MINOCA mimickers (relative to obstructive disease patients), while adjusting for the following predefined clinical indicators: age, sex, hypertension, diabetes, dyslipidemia, previous PCI, history of smoking, body mass index, left ventricle ejection fraction (LVEF), cardiogenic shock pre-PCI, year of presentation, and study site. A Cox proportional hazard model was also built to estimate relative hazard rates of 5-year mortality among patients with MINOCA as compared with MINOCA mimickers.

In a secondary analysis, a propensity score–matched sample was created using nearest-neighbor matching to assess 5-year mortality in patients with STEMI with MINOCA and MINOCA mimickers as compared with obstructive disease. Propensity scores were estimated using logistic regression on the following variables: age, sex, hypertension, diabetes, dyslipidemia, PCI history, study site, and year of admission. Matching variables were selected out of known baseline risk factors according to current literature and availability in the data; therefore, 8310 of the 8560 patients (97.1%) with complete data were included in the matching procedure. Matching was performed without replacement according to the logit of the propensity score with a 5:1 ratio of patients with MINOCA to patients with obstructive disease due to the baseline imbalance in the population sizes to assign relatively greater weight to the MINOCA cohort. To allow for imperfect matching while also enforcing similarity between matched patients, a caliper width of 0.2 standardized differences was used. One patient with MINOCA was unmatched, resulting in a matched sample of 2404 patients. Matching was conducted using the MatchIt R package, version 4.5.2 (R Project for Statistical Computing).^26^ To assess the resulting covariate balance in the matched data set, a love plot was constructed (eFigure 2 in Supplement 1). This indicated that all matched variables were well balanced after matching (since all standardized mean differences were reduced to below 0.1). Hazard ratios were estimated from the matched sample via a Cox regression model adjusting for the same variables as the unmatched model (eTable 1 in Supplement 1). The interaction term between the groups (obstructive disease or MINOCA) and PCI history was found insignificant and removed from the model. Age was modeled with a natural cubic spline with knots at the 25th, 50th, and 75th percentiles due to evidence of nonlinearity. Since this model violated the proportional hazards assumption, it was stratified by sex, and site was included in the model as a random effect. These models were then built on unmatched and matched data to assess the model’s sensitivity to modeling specifications. This procedure was repeated to estimate 5-year differences in mortality percentage among STEMI presenting with obstructive disease as compared with MINOCA mimickers. Signficance was set at a *P* value of less than .05. The confidence intervals were not adjusted for multiple comparisons. R version 4.2.3 and RStudio (R Project for Statistical Computing) in RStudio 2023 environment (Posit Software, PBC) were used for all statistical analyses. Data were analyzed from March 2003 to December 2020.

## Results

The study population included 8560 patients with STEMI, of whom 8151 (95.2%) presented with obstructive disease, 120 (1.4%) with MINOCA, and 289 (3.8%) with MINOCA mimickers. Temporal trends of STEMI diagnoses are shown in eFigure 3 in Supplement 1. Follow-up was a median (IQR) of 7.1 (3.6-10.7) years.

### Demographics and Clinical Characteristics

In the overall cohort, mean (SD) age was 62 (14) years, 30% were female (2609 participants), and 94% of the sample was non-Hispanic White (4358 participants). Hypertension was present in 5139 patients (60%), dyslipidemia in 4477 patients (54%), and smoking history in 5249 patients (64%). The median (IQR) LVEF was 50% (39%-55%), 690 patients (9%) presented with out-of-hospital cardiac arrest, and 595 patients (8%) presented with cardiogenic shock in the overall cohort (Table 1).

**Table 1.** Demographics, Clinical, and Angiographic Characteristics of Overall ST-Segment Elevation Myocardial Infarction (STEMI) Cohort and Obstructive Disease, STEMI With Nonobstructive Coronaries (MINOCA), and MINOCA Mimickers

Demographics and characteristics	Participants, No. (%)^a^			
	Overall cohort (N = 8560)	Obstructive disease (n = 8151)	MINOCA (n = 120)	MINOCA mimickers (n = 289)
Demographics				
Age, mean (SD), y	62 (14)	62 (13)	57 (16)	59 (19)
Sex				
Female	2609 (30)	2387 (29)	59 (49)	163 (56)
Male	5951 (70)	5764 (71)	61 (51)	126 (44)
Race and ethnicity				
Asian	39 (1)	37 (1)	1 (2)	1 (1)
Black	170 (4)	164 (4)	1 (2)	5 (3)
Hispanic	42 (1)	40 (1)	0	2 (1)
Non-Hispanic White	4358 (94)	4161 (93)	41 (95)	156 (94)
Other^b^	51 (1)	49 (1)	0	2 (1)
Comorbidities				
Hypertension	5139 (60)	4925 (61)	70 (58)	144 (50)
Dyslipidemia	4477 (54)	4311 (54)	56 (47)	110 (38)
Diabetes	1706 (20)	1647 (20)	22 (18)	37 (13)
Smoking history	5249 (64)	5052 (64)	63 (59)	134 (49)
Previous MI	1438 (17)	1398 (17)	18 (16)	22 (8)
Previous PCI	1685 (20)	1646 (20)	21 (18)	18 (6)
Previous stroke	223 (4)	218 (4)	3 (5)	2 (1)
Family history of CAD	3063 (48)	2934 (48)	44 (44)	85 (36)
Clinical characteristics				
Body mass index, mean (SD)^c^	29.3 (6.2)	29.3 (6.1)	28.7 (7.8)	27.7 (7.2)
SBP, mean (SD), mm Hg	140 (32)	140 (32)	141 (30)	136 (27)
DBP, mean (SD), mm Hg	84 (21)	84 (21)	84 (19)	83 (19)
Heart rate, median (IQR), bpm	77 (64-91)	76 (64-90)	82 (70-96)	89 (74-105)
LVEF, median (IQR), %	50 (39-55)	50 (40-55)	55 (50-60)	35 (28-54)
OSH transfer	3165 (65)	3040 (65)	30 (70)	95 (58)
Out-of-hospital cardiac arrest	690 (9)	670 (9)	4 (6)	16 (8)
Cardiogenic shock, pre-PCI	595 (8)	581 (8)	1 (1)	13 (6)
Killip class				
1 or 2	7134 (91)	6801 (91)	94 (95)	239 (93)
3 or 4	686 (9)	663 (9)	5 (5)	18 (7)
Laboratory markers, median (IQR)				
Troponin, ng/mL	7.8 (1.7-36.9)	8.4 (1.9-39.3)	0.6 (0.1-3.4)	1.7 (0.6-5.3)
Creatinine, mg/dL	1.0 (0.8-1.2)	1.0 (0.8-1.2)	1.0 (0.8-1.2)	0.9 (0.8-1.1)
HDL, mg/dL	37 (31-44)	37 (31-44)	43 (31-49)	41 (33-54)
LDL, mg/dL	100 (75-125)	100 (76-125)	90 (75-106)	86 (64-112)
Triglycerides, mg/dL	123 (86-174)	124 (87-175)	105 (78-147)	106 (79-135)
Total cholesterol, mg/dL	166 (138-195)	166 (138-195)	156 (136-176)	153 (124-185)
Angiographic characteristics				
Door to angiography/balloon time, median (IQR), min	94 (68-123)	94 (68-123)	89 (70-136)	97 (77-135)
Culprit vessel				
Left main	77 (1)	77 (1)	NA	NA
LAD	3176 (39)	3176 (39)	NA	NA
LCx	1182 (15)	1182 (15)	NA	NA
RCA	3499 (43)	3499 (43)	NA	NA
Multiple	107 (1)	107 (1)	NA	NA

Abbreviations: bpm, beats per minute; CAD, coronary artery disease; DBP, diastolic blood pressure; HDL, high-density lipoprotein; LAD, left anterior descending artery; LCx, left circumflex artery; LDL, low-density lipoprotein; LVEF, left ventricle ejection fraction; MI, myocardial infarction; NA, not applicable; OSH, outside hospital; PCI, percutaneous coronary intervention; RCA, right coronary artery; SBP, systolic blood pressure.

SI conversion factors: To convert creatinine to micromoles per liter, multiply by 88.4; high-density lipoprotein cholesterol to millimoles per liter, multiply by 0.259; low-density lipoprotein cholesterol to millimoles per liter, multiply by 0.259; total cholesterol to millimoles per liter, multiply by 0.259; triglycerides to millimoles per liter, multiply by 0.113; troponin to micrograms per liter, multiply by 1.0.

^a^ Percentages were computed out of all nonmissing data.

^b^ Other race included American Indian, Alaska Native, and Pacific Islander.

^c^ Body mass index is calculated as weight in kilograms divided by height in meters squared.

Compared with STEMI with obstructive disease, patients with MINOCA were younger and more often female (Table 1). The frequency of cardiovascular risk factors was similar between STEMI presenting with obstructive disease and MINOCA. STEMI presenting with MINOCA had lower incidences of cardiogenic shock at presentation, along with higher LVEF and lower peak troponin levels than obstructive disease.

Among 289 patients with MINOCA mimickers presenting with STEMI, 159 (55%) had takotsubo cardiomyopathy, 100 (35%) myocarditis, and 30 (10%) nonischemic cardiomyopathy. Compared with STEMI with obstructive disease, patients with MINOCA mimickers were more often female but similar in age (Table 1). Patients with MINOCA mimickers were less likely to have cardiovascular risk factors than patients with STEMI presenting with obstructive disease, and their LVEF and peak troponin levels were lower.

### Outcomes in STEMI Presenting With Obstructive Disease as Compared With MINOCA

**Figure 1.**
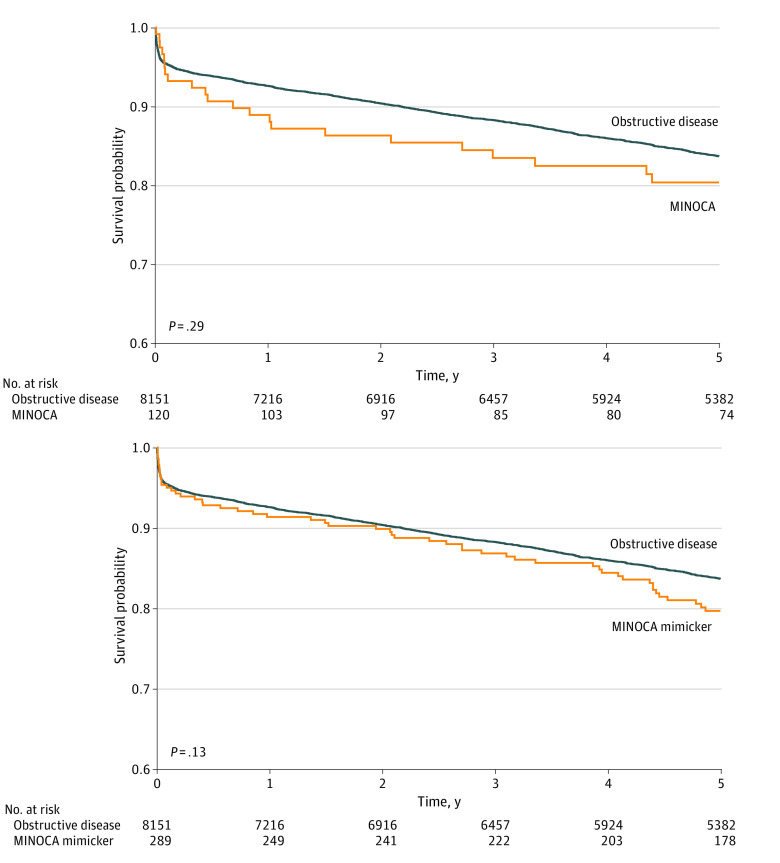
5-Year Survival Probability in ST-Segment Elevation Myocardial Infarction (STEMI) Presenting With Nonobstructive Coronaries (MINOCA) and MINOCA Mimickers in Comparison With Obstructive Disease Kaplan-Meier curves with 5-year survival probability in STEMI cases presenting with obstructive disease as compared with MINOCA and MINOCA mimickers.

**Figure 2.**
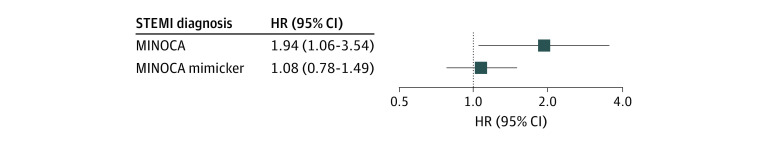
Adjusted 5-Year Mortality Risk in ST-Segment Elevation Myocardial Infarction (STEMI) Presenting With Nonobstructive Coronaries (MINOCA) and MINOCA Mimickers in Comparison With Obstructive Disease Five-year mortality risk in adjusted models for age, sex, hypertension, diabetes, dyslipidemia, percutaneous coronary intervention history, history of smoking, body mass index, left ventricle ejection fraction, cardiogenic shock at presentation, study site, and year of admission. Reference is STEMI obstructive disease.

Compared with STEMI presenting with obstructive disease, MINOCA cases were less frequently discharged receiving aspirin; purinergic receptor P2Y, G-protein coupled, 12 protein (P2Y12) inhibitors; statins; β-blockers; and angiotensin-converting enzyme inhibitors (ACEI) or angiotensin receptor blockers (ARB) (Table 2). In-hospital mortality, 1-year mortality, and 1-year MACE risk were similar in STEMI presenting with obstructive disease as compared with MINOCA. Survival probability at 5 years was similar in STEMI presenting with MINOCA (20 participants [18%]) as compared with obstructive disease (1228 participants [16%]; χ^2^_1_ = 1.1; log-rank *P* = .29) (Figure 1A). In multivariable adjusted analysis, the 5-year mortality hazard risk in STEMI presenting with MINOCA was 1.93 times higher (95% CI, 1.06-3.53) than obstructive disease (Figure 2).

**Table 2.** Outcomes of ST-Segment Elevation Myocardial Infarction (STEMI) Presenting With Obstructive Disease Compared With STEMI Presenting With Nonobstructive Coronaries (MINOCA)

Outcomes	Participants, No. (%)^a^		
	Obstructive disease (n = 8151)	MINOCA (n = 120)	*P* value
Length of stay, mean (IQR), d	2 (2-4)	2 (1-3)	<.001
Medications at discharge			
Aspirin	6939 (95)	47 (75)	<.001
P2Y12 inhibitor	6042 (91)	20 (31)	<.001
Statin	6708 (93)	36 (57)	<.001
β-Blocker	6671 (93)	42 (67)	<.001
ACEI/ARB	5847 (72)	52 (53)	<.001
Mortality			
In-hospital death	325 (4)	2 (2)	.67
1-y MACE	408 (13)	8 (23)	.09^b^
1-y Death	584 (7)	13 (12)	.12^b^
5-y Death	1228 (16)	20 (18)	.29^b^

Abbreviations: ACEI, angiotensin-converting enzyme inhibitors; ARB, angiotensin receptor blocker; MACE, major adverse cardiovascular event; P2Y12, purinergic receptor P2Y, G-protein coupled, 12 protein.

^a^ Percentages were computed out of all nonmissing data.

^b^ Log-rank *P.*

### Outcomes in STEMI Presenting With Obstructive Disease as Compared With MINOCA Mimickers

Compared with STEMI presenting with obstructive disease, MINOCA mimicker cases were less frequently discharged receiving aspirin, P2Y12 inhibitors, statins, β-blockers, and ACEIs or ARBs (Table 3). In-hospital mortality, 1-year mortality, and 1-year MACE risk were similar in STEMI presenting with obstructive disease as compared with MINOCA mimickers. Survival probability at 5 years was similar in STEMI presenting with MINOCA as compared with obstructive disease (Figure 1B). At 5-year follow-up, mortality in STEMI presenting with obstructive disease (1228 participants [16%]) was similar to MINOCA mimickers (52 participants [18%]; χ^2^_1_ = 2.3; log-rank *P* = .13). In multivariable adjusted analysis, the 5-year mortality risk in STEMI presenting with MINOCA mimickers was similar to obstructive disease (HR, 1.08; 95% CI, 0.79-1.49) (Figure 2).

**Table 3.** Outcomes of ST-Segment Elevation Myocardial Infarction (STEMI) Presenting With Obstructive Disease Compared With STEMI Presenting With Nonobstructive Coronaries (MINOCA) Mimickers

Outcome	Participants, No. (%)		*P* value
	Obstructive disease (n = 8151)	MINOCA mimickers (n = 120)	
Length of stay, mean (IQR), d	2 (2-4)	2 (1-5)	.81
Medications at discharge			
Aspirin	6939 (95)	101 (53)	<.001
P2Y12 inhibitor	6042 (91)	7 (4)	<.001
Statin	6708 (93)	64 (34)	<.001
β-Blocker	6671 (93)	121 (64)	<.001
ACEI/ARB	5847 (72)	156 (60)	.003
Mortality			
In-hospital death	325 (4)	12 (4)	.89
1-y MACE	408 (13)	15 (14)	.71^a^
1-y Death	584 (7)	23 (8)	.51^a^
5-y Death	1228 (16)	52 (18)	.13^a^

Abbreviations: ACEI, angiotensin-converting enzyme inhibitors; ARB, angiotensin receptor blocker; MACE, major adverse cardiovascular event; P2Y12, purinergic receptor P2Y, G-protein coupled, 12 protein.

^a^ Log-rank *P.*

### Outcomes in STEMI Presenting With MINOCA as Compared With MINOCA Mimickers

Compared with STEMI presenting with MINOCA, MINOCA mimicker cases were less frequently discharged receiving aspirin, P2Y12 inhibitors, and statins, but no significant difference was observed in β-blockers and ACEIs or ARBs (eTable 2 in Supplement 1). In-hospital, 1-year, and 5-year mortality risks and 1-year MACE events were similar in STEMI presenting with MINOCA as compared with MINOCA mimickers. In multivariable adjusted analysis, the 5-year mortality risk in STEMI presenting with MINOCA was similar to MINOCA mimickers (HR, 1.65; 95% CI, 0.68-4.01).

### Matched Cohort Secondary Analysis

In secondary analysis of the matched cohort, both patients with MINOCA and MINOCA mimickers had higher 5-year mortality risk compared with obstructive disease (χ^2^_1_ = 7.3; log-rank *P* = .01 and χ^2^_1_ = 5.8; *P* = .02, respectively). In multivariable adjusted analysis of the matched cohort, the 5-year mortality risk in STEMI presenting with obstructive disease was similar to MINOCA (HR, 1.93; 95% CI, 0.93-4.01) and MINOCA mimickers (HR, 1.31; 95% CI, 0.89-1.92).

### CMRI Findings

The frequency of CMRI use in STEMI cases presenting with MINOCA was 30 for site 1, 1 for site 2, and 0 for site 3. Among STEMI cases presenting with MINOCA, the number of MINOCA cases with diagnoses were significantly higher at 76% (34 of 45 patients) at site 1, where CMR was routinely used, compared with site 2 at 42% (8 of 19 patients) and site 3 at 57% (32 of 56 patients; *P* for trend = .03). At site 1, CMRI was performed in 67% of MINOCA cases (30 of 45 patients). Sensitivity analysis evaluating 5-year mortality among the CMRI-diagnosed MINOCA cases was similar to the overall cohort. Mortality at 5 years in STEMI cases presenting with obstructive disease was similar to MINOCA (χ^2^_1_ = 1.4; *P* = .24). At site 1, CMRI was performed in 49% of MINOCA mimicker cases (83 of 169 patients). By contrast, only 3 CMRI were performed among MINOCA mimicker cases at sites 2 and 3. Among the 83 patients with MINOCA mimickers who underwent CMRI at site 1, 37 had takotsubo cardiomyopathy, 42 had myocarditis, and 3 had nonischemic cardiomyopathy.

## Discussion

In this large multicenter prospective cohort of 8560 consecutive patients with STEMI, several observations advance our understanding of MINOCA and MINOCA mimickers: (1) MINOCA was present in 1.4% and MINOCA mimickers in 3.8% of consecutive patients presenting with STEMI; (2) MINOCA and MINOCA mimickers represent conditions as lethal as patients with obstructive disease in short-term and long-term follow-up; (3) 5-year mortality hazard risk was higher in MINOCA than obstructive disease; (4) MINOCA and MINOCA mimickers were less likely to receive cardiac medications at hospital discharge compared with obstructive disease; and (5) CMRI was useful to establish an underlying diagnosis among patients with MINOCA and MINOCA mimickers.

In our cohort of consecutive patients with STEMI, 120 patients (1.4%) presented with MINOCA and 289 (3.8%) with MINOCA mimicker diagnoses. These findings are consistent with Gue et al^27^ who reported 110 cases (4.4%) using the ESC definition, which combined MINOCA and MINOCA mimickers in a smaller cohort of consecutive STEMI cases in the United Kingdom. In their study, the 1-year mortality was 4.5%, which was lower than our 1-year mortality of 12% in MINOCA and 8% in MINOCA mimicker cases.^27^ We also observed an 18% mortality at 5-year follow-up in both MINOCA and MINOCA mimicker cases presenting with STEMI. By contrast, Andersson et al^20^ reported an 8% mortality risk in patients who presented with MINOCA in a single-center study in Denmark among 4793 consecutive patients with STEMI during a 2.6-year follow-up. The higher rates observed in our study may be due in part to the longer follow-up time, the overall higher death rates in this US midwest cohort, and the inclusion of normal cardiac biomarkers in the MINOCA cohort in the Denmark study.

Only 2 prior studies^19,20^ that we know of have evaluated outcomes in STEMI patients presenting with MINOCA as compared with obstructive disease with conflicting findings. Importantly, neither of these studies reported whether MINOCA mimickers were included in the MINOCA group, and both studies included patients with normal cardiac biomarkers. The HORIZONS-AMI trial^19^ evaluated the safety and efficacy of bivalirudin, and in a post hoc analysis of 3602 patients with STEMI, including 127 (3.5%) with no obstructive disease, at 3-year follow-up, MACE risk was lower in patients without obstructive disease as compared with patients with obstructive disease (mortality risk was not reported).^19^ The lower MACE risk in the HORIZONS-AMI post hoc analysis was likely associated with the lower threshold used to define obstructive coronary artery disease (>30% stenosis) and the lack of elevated ischemia biomarkers in nearly 50% of patients. Furthermore, in the Denmark study by Andersson et al,^20^ the subgroup of patients with normal coronary arteries and elevated troponin had a 2.65-fold higher risk of death than patients with obstructive disease. Similarly, in our study, we found a 1.93-fold higher risk of 5-year mortality in patients with MINOCA as compared with obstructive disease and also found a similar risk in MINOCA mimickers as compared with obstructive disease.

MINOCA poses a dilemma for clinicians given the multitude of diagnoses that underlie this enigmatic syndrome. Our results suggest that there are remarkable differences in STEMI cases presenting with obstructive disease as compared with MINOCA and MINOCA mimickers. The differences in risk profile, treatment, and outcomes in MINOCA and MINOCA mimickers support the differentiation made by the recent AHA guidelines between coronary (MINOCA) and noncoronary (MINOCA mimicker) causes of MI without a culprit artery.^5^ In this study, the use of CMRI was infrequent in STEMI cases presenting with MINOCA. We demonstrate that at site 1, where there was a standardized protocol to perform CMRI for MINOCA cases, CMRI substantially improved the ability to identify the underlying diagnosis in 76% of MINOCA cases. These results are consistent with and extend the findings of the HARP study,^28^ which demonstrated the utility of multimodality imaging to diagnose the underlying diagnosis of MINOCA events in a cohort predominantly composed of patients with NSTEMI. Coronary intravascular imaging is another important tool in determining the underlying diagnosis of MINOCA,^28^ but it was not used at any of the study sites. Our findings support the need for standardized algorithms in the diagnosis of STEMI presenting with MINOCA that includes the use of CMRI in all cases.^29,30^

Notably, we documented that patients with MINOCA and MINOCA mimickers presenting with STEMI were less likely to be prescribed cardiac medications upon discharge compared with obstructive disease, similar to the treatment disparities reported in MINOCA cohorts presenting with NSTEMI.^31,32^ Patients with MINOCA likely receive suboptimal treatment for several reasons: (1) the popular misconception that MINOCA cases are often false positive troponins; (2) lack of recognition that a MINOCA event is associated with poor outcomes; (3) diversity in the range of underlying diagnoses that cause MINOCA (plaque disruption, coronary vasospasm, and embolism); and (4) limited knowledge regarding effective treatments for MINOCA. There is growing evidence that the nonuse of statins and renin-angiotensin-aldosterone system (RAAS) inhibitors is associated with a 2-fold higher mortality risk in patients with MINOCA.^33,34^ Yet our study demonstrates that work remains to be done, given the significantly lower use of RAAS inhibitors and statins in STEMI presenting with MINOCA. Therefore, we suggest including MINOCA in STEMI quality metrics.

### Limitations

This study has limitations common to observational studies. Angiographic images were not evaluated by a core laboratory; however, each case of STEMI without a culprit on angiography was reviewed by the principal investigators at each site, including all available angiograms. Follow-up was performed with the use of NDI and electronic medical records at all the sites, which was a limitation as this can result in biased survival analysis. The lack of a standardized protocol at all sites and resulting underuse of CMRI and nonuse of intravascular imaging greatly limited our ability to further determine the underlying diagnosis of MINOCA cases. However, the low utilization of multimodality imaging in our study is consistent with clinical experience in the US. Furthermore, granular data on cause of death were not available. Because of these limitations, this study should be viewed as hypothesis-generating to stimulate the need for additional investigations.

## Conclusions

In contrast to the general prevailing sentiment, STEMI due to MINOCA and MINOCA mimickers are lethal as obstructive disease. We demonstrate that the 5-year mortality risk is 1.93-fold higher in MINOCA as compared with obstructive disease. The current underutilization of CMRI and intravascular imaging restricts our ability to explore the underlying diagnoses of MINOCA and MINOCA mimickers, which could play a crucial role in guiding future management and prognosis.

## Notes

Supplement 1. **eTable 1.** Summary of Modeling Variables in the Matched Data Set **eTable 2.** Outcomes of STEMI Presenting With MINOCA Compared With MINOCA Mimickers **eFigure 1.** Study Flow Chart **eFigure 2.** Love Plot of Covariate Balance in the Matched and Unmatched Dataset **eFigure 3.** Temporal Trends in STEMI Diagnosis

Supplement 2.
Data Sharing Statement
