# Supplementary material for: Mortality in ST-Segment Elevation Myocardial Infarction With Nonobstructive Coronary Arteries and Mimickers
Source: JAMA Netw Open. 2023 Nov 16;6(11):e2343402. doi: 10.1001/jamanetworkopen.2023.43402 (PMC10654797; doi:10.1001/jamanetworkopen.2023.43402)
Supplement: Supplement 1. — eTable 1. Summary of Modeling Variables in the Matched Data Set eTable 2. Outcomes of STEMI Presenting With MINOCA Compared With MINOCA Mimickers eFigure 1. Study Flow Chart eFigure 2. Love Plot of Covariate Balance in the Matched and Unmatched Dataset eFigure 3. Temporal Trends in STEMI Diagnosis [file jamanetwopen-e2343402-s001.pdf]

Supplemental Online Content

Quesada O, Yildiz M, Henry TD, et al. Mortality in ST-segment elevation myocardial infarction with nonobstructive coronary arteries and mimickers. *JAMA Netw Open*. 2023;6(11):e2343402. doi:10.1001/jamanetworkopen.2023.43402

eTable 1. Summary of Modeling Variables in the Matched Data Set
eTable 2. Outcomes of STEMI Presenting With MINOCA Compared With MINOCA-Mimicker
eFigure 1. Study Flow Chart
eFigure 2. Love Plot of Covariate Balance in the Matched and Unmatched Dataset
eFigure 3: Temporal Trends in STEMI Diagnosis

This supplemental material has been provided by the authors to give readers additional information about their work.

© 2023 Quesada O et al. *JAMA Network Open.*

eTable 1. **Summary of Modeling Variables in the Matched Data Set**

Variable Characteristics	Overall Cohort (n = 2402)	Obstructive Disease (n = 1998)	MINOCA (n = 120)	MINOCA-Mimicker (n = 286)
Age, y, mean (SD)	59 (15)	59 (14)	57 (16)	59 (19)
Female, n (%)	1341 (56)	1121 (56)	59 (49)	161 (56)
Year of presentation	2012 (2009, 2016)	2012 (2009, 2016)	2013 (2010, 2016)	2012 (2009, 2016)
Hypertension, n (%)	1339 (56)	1125 (56)	70 (58)	144 (50)
Dyslipidemia, n (%)	1037 (43)	871 (44)	56 (47)	110 (38)
Diabetes, n (%)	394 (16)	336 (17)	22 (18)	36 (13)
Smoking history, n (%)	1437 (63)	1241 (65)	63 (59)	133 (49)
BMI, kg/m2, mean (SD)	29 (7)	30 (7)	29 (8)	28 (7)
Previous PCI, n (%)	351 (15)	312 (16)	21 (18)	18 (6)
Cardiogenic shock, pre-PCI, n (%)	159 (8)	146 (8)	1 (1)	12 (6)
LVEF, %, median (IQR)	50 (38, 58)	50 (40, 58)	55 (50, 60)	35 (29, 55)
5-year death, n (%)	317 (14)	246 (13)	20 (18)	51 (18)

BMI, body mass index; PCI, percutaneous coronary intervention; LVEF, left ventricle ejection fraction.

© 2023 Quesada O et al. *JAMA Network Open.*

eTable 2. Outcomes of STEMI Presenting With MINOCA Compared With MINOCA-Mimicker

Outcomes	MINOCA N = 120	MINOCA-Mimicker N = 289	p-value
**Length of stay,** days, mean (IQR)	2 (1, 3)	2 (1, 5)	0.006
**Medications at discharge**			
Aspirin, n (%)	47 (75)	101 (53)	0.009
P2Y12 inhibitor, n (%)	20 (31)	7 (4)	<0.001
Statin, n (%)	36 (57)	64 (34)	0.008
Beta blocker, n (%)	42 (67)	121 (64)	0.9
ACEI/ARB, n (%)	52 (53)	156 (60)	0.09
**Mortality**			
In-hospital death, n (%)	2 (2)	12 (4)	0.37
1-year MACE, n (%)	8 (23)	15 (14)	0.25*
1-year death, n (%)	13 (12)	23 (8)	0.46*
5-year death, n (%)	20 (18)	52 (18)	1.0*

Abbreviations: P2Y12, purinergic receptor P2Y, G-protein coupled,12 protein; ACEI, Angiotensin-converting enzyme inhibitors; ARB, angiotensin receptor blocker; MACE, major adverse cardiovascular events.
*Log-rank *P* value.

© 2023 Quesada O et al. *JAMA Network Open.*

eFigure 1. Study Flow Chart

The study population was derived from a multi-center, prospective Midwest STEMI consortium registry.

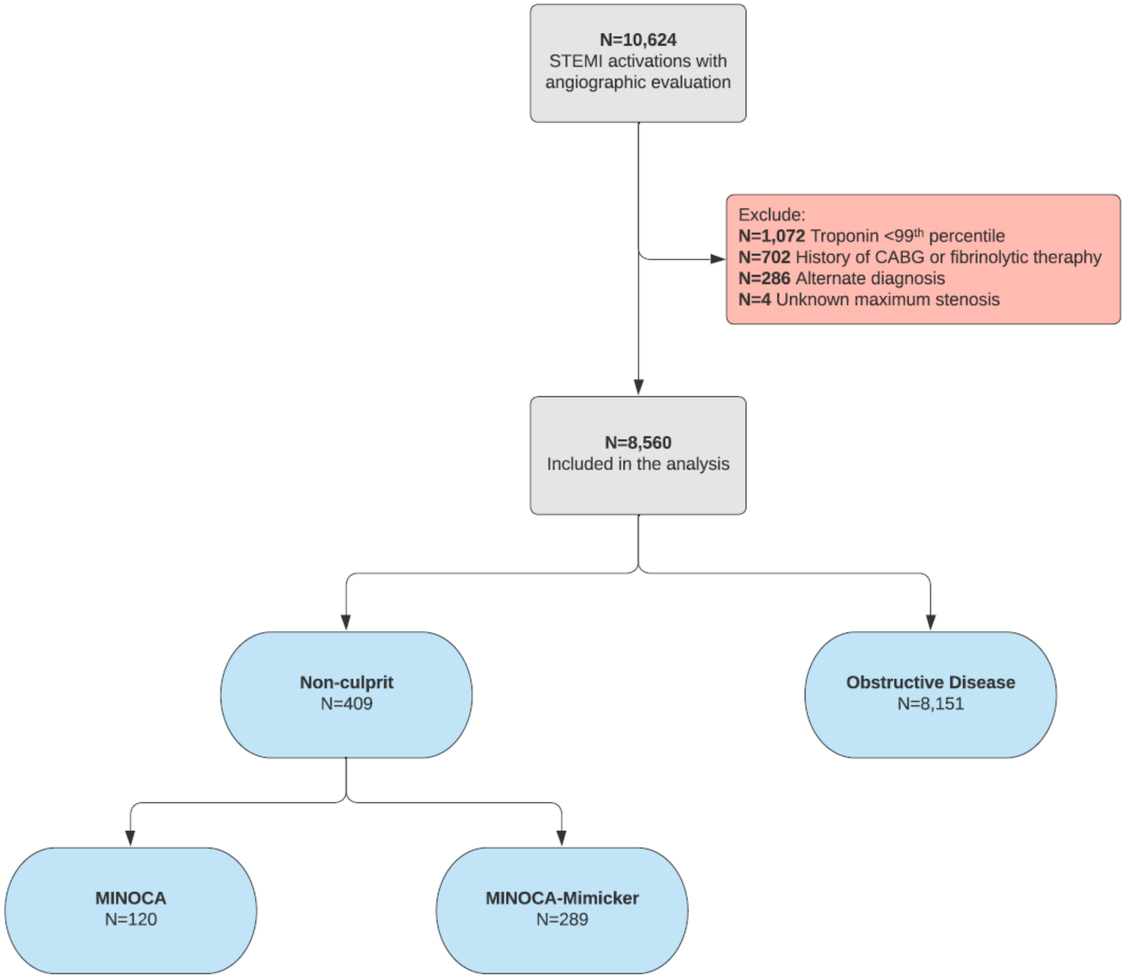

© 2023 Quesada O et al. *JAMA Network Open.*

eFigure 2. Love Plot of Covariate Balance in the Matched and Unmatched Dataset

Love plot demonstrates covariate balance in the matched and unmatched dataset.

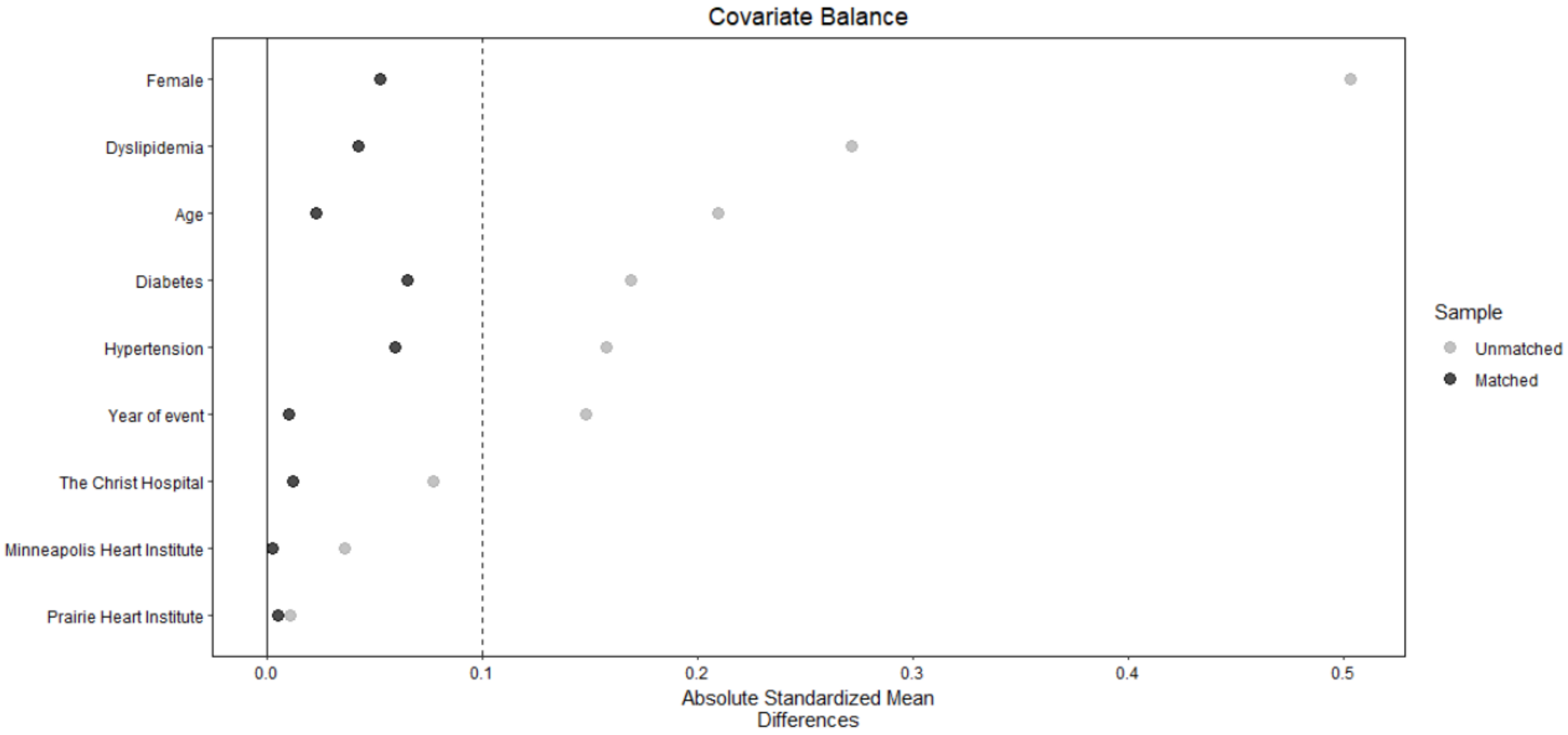

© 2023 Quesada O et al. *JAMA Network Open.*

eFigure 3: Temporal Trends in ST-Segment Elevation Myocardial Infarction (STEMI) by Diagnosis

Trends in the Proportion of ST-Elevation Myocardial Infarction (STEMI) Cases Presenting With Obstructive Disease, STEMI Presenting With Nonobstructive Coronary Arteries (MINOCA), and MINOCA-Mimickers by Year of Presentation.

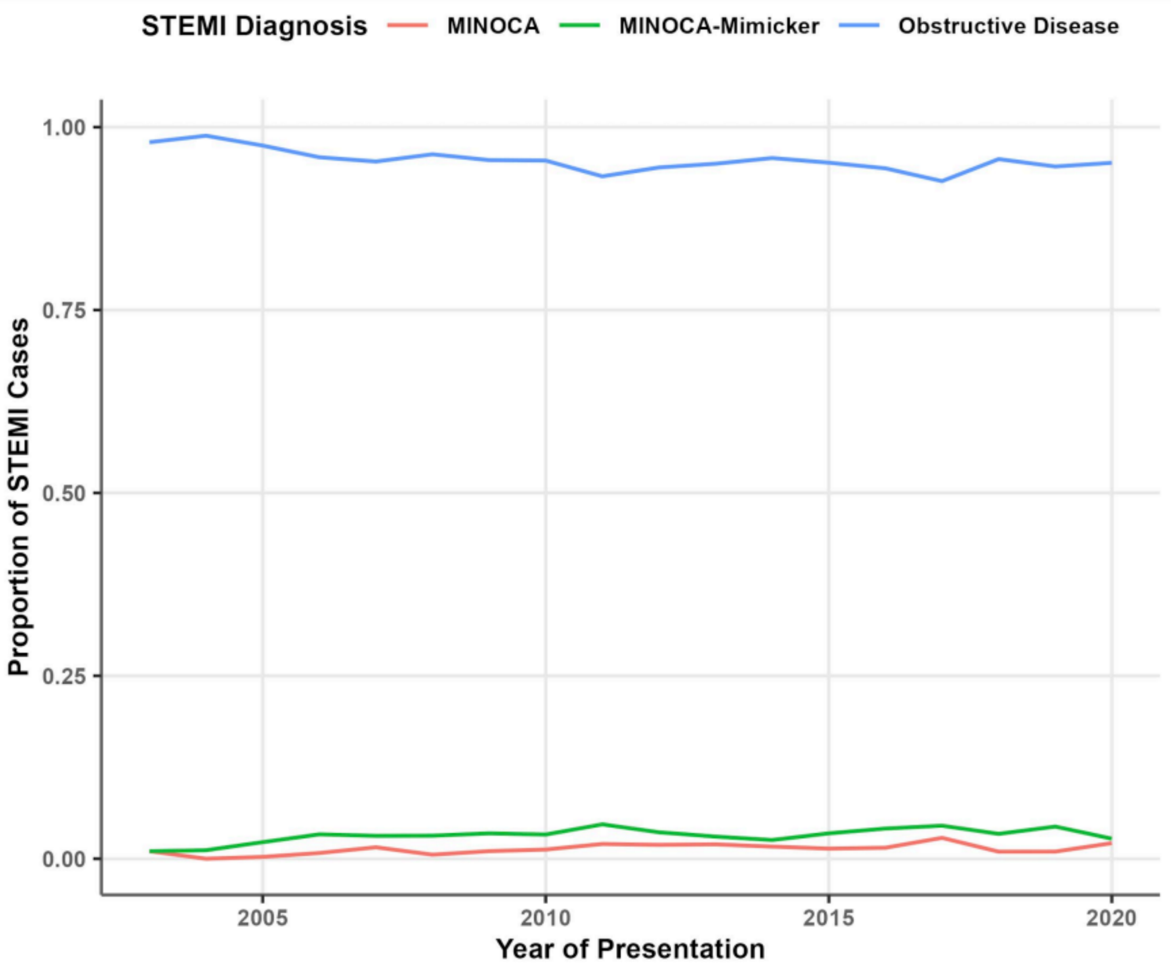

© 2023 Quesada O et al. *JAMA Network Open.*
