# Supplementary material for: Mortality in ST-Segment Elevation Myocardial Infarction With Nonobstructive Coronary Arteries and Mimickers
Source: JAMA Netw Open. 2023 Nov 16;6(11):e2343402. doi: 10.1001/jamanetworkopen.2023.43402 (PMC10654797; doi:10.1001/jamanetworkopen.2023.43402)
Supplement: Supplement 2. — Data Sharing Statement [file jamanetwopen-e2343402-s002.pdf]

Data Sharing Statement

Quesada. Mortality in ST-Segment Elevation Myocardial Infarction With Nonobstructive Coronary Arteries and Mimickers. *JAMA Netw Open*. Published November 16, 2023. doi:10.1001/jamanetworkopen.2023.43402

Data
Data available: No

Additional Information
Explanation for why data not available: Will provide after acceptance
